# Lobar Mucus Plugging Reflecting Central Bronchial Tumoral Obstruction: A Case Report

**DOI:** 10.7759/cureus.48874

**Published:** 2023-11-15

**Authors:** Asma Bensliman, Denis Tack

**Affiliations:** 1 Radiology, Université libre de Bruxelles (ULB), Brussels, BEL; 2 Radiology, Centre Hospitalier EpiCURA - site de Ath, Ath, BEL

**Keywords:** pulmonary carcinoid tumor, mucoid impaction, bronchorrhea, bronchocele, lobar mucus plugging, bronchial obstruction

## Abstract

Early diagnosis of bronchopulmonary carcinoid tumors is crucial as the surgical excision is the main treatment and determines the prognosis. We present the case of a 66-year-old heavy-smoker man who had started to complain about a cough a few months ago. We diagnosed him with an endobronchial mass on a chest computed tomography scan and lobar bronchoceles resulting from mucus plugging distal to the tumor obstruction. These findings were retrospectively visible on the previous chest radiograph that had initially been interpreted as non-contributary.

## Introduction

Bronchopulmonary carcinoid tumors represent less than 2% of lung cancer [1]. Early diagnosis is crucial as the surgical excision is the main treatment and determines the prognosis [2]. Carcinoid tumors are central and perihilar and the majority originate from a lobar or segmental bronchus [3]. In early stages, patients may only show obstructive signs such as bronchocele due to lobar mucus plugging [3]. It is therefore essential to have knowledge about this sometimes subtle radiologic sign.

## Case presentation

**Figure 1:**
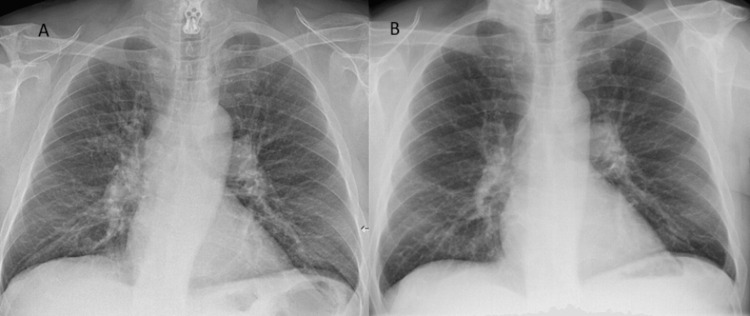
Posteroanterior chest X-rays realized for cough (a) and three years ago (b).

A 66-year-old heavy-smoker man had started to complain about a cough a few months ago. His physician prescribed him a chest X-ray that was initially misinterpreted as normal (Figure 1A). He also had a normal chest X-ray three years ago for fever (Figure 1B). 

**Figure 2:**
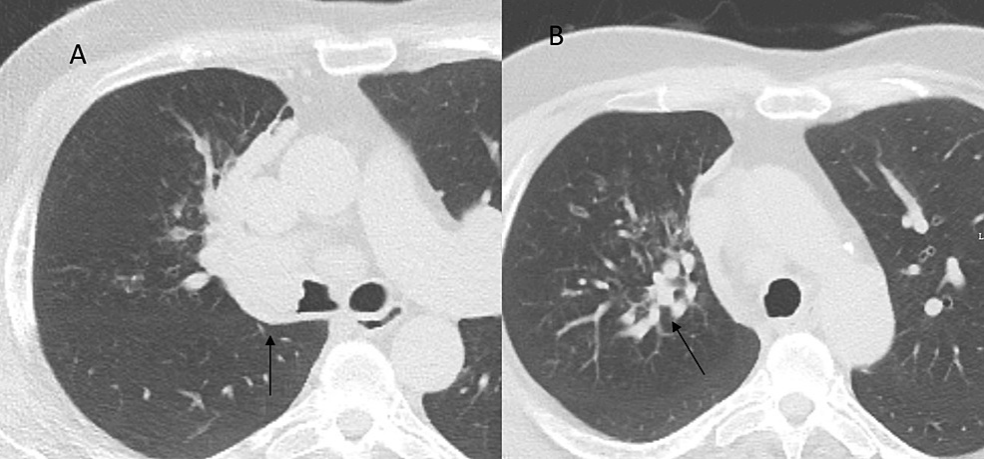
Chest CT showing an endobronchial mass (A) and bronchocele resulting from mucus plugging distal to the tumor obstruction in segmental and subsegmental bronchi of the right upper lobe (B). We clearly see enlarged and filled bronchi in the right upper lobe compared to the contralateral side.

Computed tomography (CT) performed a few days later showed an endobronchial mass in the right upper lobe (RUL) and bronchoceles resulting from mucus plugging distal to the tumor obstruction (Figures 2A, 2B). Mediastinal lymphadenopathy was also described.

**Figure 3:**
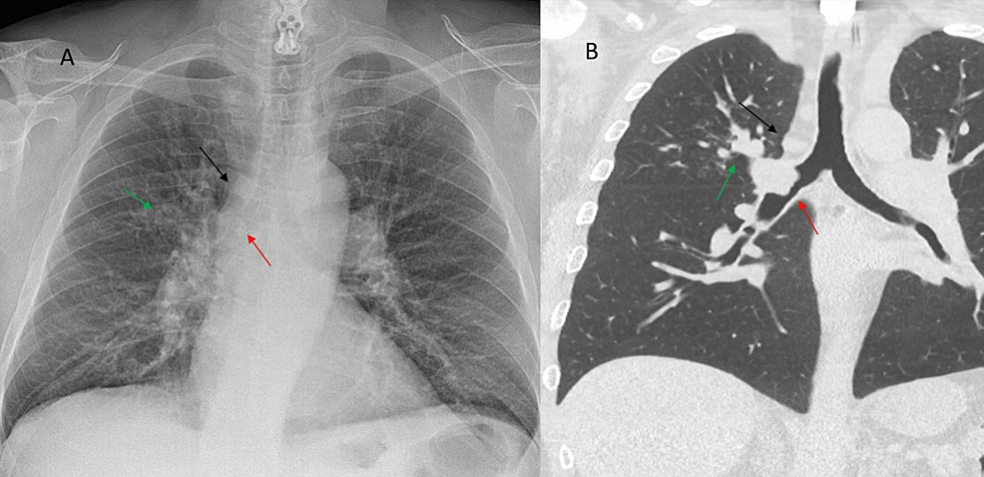
Last chest X-ray realized (A) and corresponding CT section (B). The red arrow shows the endobronchial mass. The black arrow shows the widening of the right paratracheal stripe and the mediastinal adenomegaly. The green arrow shows the distal mucus plugging in the right upper lobe.

Retrospectively, these findings were visible on the chest X-ray. We can first notice a widening of the right paratracheal stripe (RPS) with a round opacity that corresponds to the mediastinal adenomegaly seen in the corresponding CT section. We also noticed enlarged bronchovascular structures with branching opacities. These appeared to correspond to the bronchoceles due to the mucus plugging in the RUL on CT. Finally, the endobronchial tumor can also be seen on the chest X-ray as a well-defined opacity herniating in the right mainstem bronchus (Figures 3A, 3B).

All these anomalies were clearly absent on the chest X-ray that was done three years ago (Figure 1B). The patient underwent a bronchoscopy with microbiopsies which demonstrated a bronchial carcinoid tumor, classified as neuroendocrine neoplasm of the lung.

## Discussion

The Fleischner Society of glossary defines the bronchocele as a bronchial dilatation due to retained secretions (mucus impaction), usually caused by a proximal congenital or acquired obstruction, with or without an underlying bronchial dilatation [4]. The two main mechanisms leading to an excessive accumulation of mucus are excessive or abnormal production of mucus and impaired drainage [5]. There are many different causes of mucus plugging that can be divided into two groups of conditions. First, those that involve bronchial obstruction like neoplasms, foreign bodies, congenital bronchial atresia, broncholithiasis, and tuberculosis. Secondly, those that do not involve bronchial obstruction, like asthma, cystic fibrosis, and allergic bronchopulmonary aspergillosis (ABPA) [5]. Cystic fibrosis is a genetic disorder characterized by a defect in the chloride transporter. These patients are more likely to have ABPA [6].

Besides neoplasms, bronchocele due to mucus plugging in a unilobar distribution often results from ABPA. The mucoid impaction usually involves the upper lobes and, in a few cases, the lower lobes only [6]. ABPA is also characterized by high-attenuation mucus in up to 20% of patients [5,6]. Congenital bronchial atresia is another rare cause of unilobar mucoid impaction and affects mostly the apical posterior segment of the left upper lobe [7]. 

CT chest is of course the investigation of choice for mucus plugging, but indirect signs on chest X-rays can be appreciated as described in our case. Branching opacities and “finger in glove” appearance, characterized by branching tubular or fingerlike opacities that often originate from the hila and are directed peripherally, are classical findings [5,8]. Another important sign that we present in this case is the widening of the RPS. The width of the RPS in normal subjects ranges from 1 to 4 millimeters. The RPS is formed by the trachea, the mediastinal connective tissue and its content, and paratracheal pleura. Each of these tissues must be considered in the evaluation of the RPS widening [9].

## Conclusions

Bronchocele resulting from mucus plugging is an essential sign to detect on a chest X-ray, especially when unilobar to rule out an underlying neoplastic lesion, such as bronchopulmonary carcinoid in our case, and allow further investigations.

Through this case report, we hope to bring attention to this indirect sign and avoid delayed diagnosis of a bronchopulmonary tumor that requires early management.
